# Hyperspectral Imaging for Quality Assessment of Processed Foods: A Case Study on Sugar Content in Apple Jam

**DOI:** 10.3390/foods14213585

**Published:** 2025-10-22

**Authors:** Danila Lissovoy, Alina Zakeryanova, Rustem Orazbayev, Tomiris Rakhimzhanova, Michael Lewis, Huseyin Atakan Varol, Mei-Yen Chan

**Affiliations:** 1 School of Engineering and Digital Sciences, Nazarbayev University, Astana 010000, Kazakhstan; 2 Institute of Smart Systems and Artificial Intelligence, Nazarbayev University, Astana 010000, Kazakhstan; tomiris.khalimova@nu.edu.kz (T.R.);; 3 Department of Biomedical Sciences, School of Medicine, Nazarbayev University, Astana 010000, Kazakhstan

**Keywords:** hyperspectral imaging, apple jam, sugar concentration, machine learning, Support Vector Machine, XGBoost, ResNet, non-destructive analysis, food authenticity, food adulteration

## Abstract

Apple jam is a widely used all-season product. The quality of the jam is closely related to its sugar concentration, which affects its taste, texture, shelf life, and legal compliance with production requirements. Although traditional methods for measuring sugar, such as titration, enzymatic methods, and chromatography, are accurate, they are also invasive, destructive, and unsuitable for rapid screening. This study investigates a non-destructive and non-invasive alternative method that uses hyperspectral imaging (HSI) in combination with machine learning to estimate the sugar content in processed apple products. Eight cultivars were selected from the Central Asian region, recognized as the origin of apples and known for its rich diversity of apple cultivars. A total of 88 jam samples were prepared with sugar concentrations ranging from 25% to 75%. For each sample, several hyperspectral images were obtained using a visible-to-near-infrared (VNIR) camera. The acquired spectral data were then processed and analyzed using regression models, including the support vector machine (SVM), eXtreme gradient boosting (XGBoost), and a one-dimensional residual network (1D ResNet). Among them, ResNet achieved the highest prediction accuracy of R^2^ = 0.948. The results highlight the potential of HSI and machine learning for a fast, accurate, and non-invasive assessment of the sugar content in processed foods.

## 1. Introduction

Apples are one of the most widely consumed and cultivated fruits worldwide, and serve as a good source of vitamins and antioxidants. Regular consumption of fruits, including apples, has been associated with health benefits such as a lower risk of developing asthma and cardiovascular diseases, and some types of cancers [1]. The origins of this nutritionally valuable fruit can be traced back to Central Asia, which is widely recognized as the primary center of genetic diversity, due to the presence of the wild ancestor of modern apple cultivars Malus sieversii [2]. The region’s unique ecological [3] conditions and long history of cultivation have contributed to an exceptionally rich apple gene pool, featuring diverse fruit sizes, shapes, colors, textures, and flavors. This natural variability makes Central Asian apples an ideal subject for studies focused on this area [2].

Apples are seasonal fruits. To prolong their availability beyond the harvesting season, they are frequently preserved in the form of jams, jellies, and marmalades. Apple jam is a processed product made by boiling apple puree with sugars, as sucrose or fructose syrup [4]. Over the years, this traditional practice of fruit preservation has developed into a worldwide industry. Increasing demand for natural, fruit-based products has fueled the fruit jam, jelly, and preserves market, which was worth USD 1.2 billion in 2024 and is predicted to reach USD 1.8 billion by 2033 [5].

Accurate sugar concentration in apple jam is crucial for maintaining quality, ensuring safety, and complying with legal requirements. According to the Codex Alimentarius, the total soluble solids (TSSs) must be at least 60–65% [4]. Insufficient sugar can compromise product quality through spoilage, while excessive intake poses health risks such as weight gain and obesity [6], highlighting the need for precise measurement methods [7,8]. Traditional methods for analyzing sugar content, such as titration [9,10], chromatography (e.g., high-performance liquid chromatography [11,12], gas chromatography–mass spectrometry [13]), and enzymatic assays [14], are highly accurate but also destructive, time-consuming, and require well-trained personnel and specialized laboratory conditions [15]. Faster, non-destructive alternatives, such as refractometry [16], near-infrared spectroscopy [17,18], and Fourier transform infrared spectroscopy [19], are commonly used; however, they lack spatial resolution [20], which renders them less effective in industrial conditions where real-time monitoring is necessary.

Hyperspectral imaging (HSI), especially when combined with machine learning techniques, offers a non-destructive and cost-effective alternative that requires minimal sample preparation and does not necessitate a sterile laboratory setting [15]. HSI has gained popularity in various fields, including ecology [21,22], medicine [23], and food quality control [24] due to its high precision and capacity to automatically analyze large amounts of data.

In the food industry, HSI is used to assess physical and chemical quality characteristics in products such as meat [25,26], tea [27], and coffee [28], as well as to monitor the health of crops [24]. These applications improve quality control while also reducing operational costs through process automation and early detection of irregularities.

While existing research studies have demonstrated the capability of HSI in assessing the quality of fruits and vegetables, including moisture, firmness, and ripeness, its application in processed food products remains underexplored. One frequently targeted parameter is soluble solids content (SSC), which refers to the concentration of sugars, acids, and other dissolved compounds that influence taste, maturity, and commercial value. As summarized in Table 1, many studies have successfully employed HSI in combination with machine learning techniques, including classical algorithms and deep learning models, to predict SSC and related attributes in the raw product. For example, Rady et al. [29] applied partial least squares regression (PLSR) and neural networks to evaluate glucose levels in potatoes, while Yun et al. [30] utilized a 1D ResNet to enhance the predictive accuracy of state-of-the-art techniques for tomato firmness. Similar approaches have been applied to grapes [31], kiwis [32], strawberries [33], peaches [34], and apples [35,36,37], demonstrating high predictive accuracy.

Although HSI is well established in raw produce quality assessment, its use for processed fruit products remains underexplored [38,39]. Processed foods introduce additional complexity due to heterogeneous composition and visual noise, which makes non-destructive quality assessment more challenging.

The literature review did not identify any studies that investigated an application of hyperspectral imaging combined with machine learning to estimate sugar content in a processed food product, such as apple jam. Jam is defined as a product brought to a suitable consistency, made from whole fruit, pieces of fruit, unconcentrated and/or concentrated fruit pulp or fruit puree of one or more kinds of fruit, mixed with sweetening foodstuffs with or without the addition of water [4]. This research addresses this gap by applying HSI, coupled with machine learning models, to assess the sugar content of apple jam, a commonly consumed processed fruit product. In this context, machine learning enables the automatic discovery of patterns in the hyperspectral data, allowing sugar concentration to be predicted from image information without destructive testing and makes the following main contributions:

- Release of an open-source, well-structured, annotated hyperspectral image dataset of apple jam prepared from 8 apple cultivars native to Central Asia, the primary center of apple genetic diversity, across eleven sugar levels.
- Acquisition of 1760 annotated hyperspectral images under controlled lighting, angle, and distance conditions using a visible to near-infrared (VNIR) hyperspectral camera, to provide a standardized and reproducible basis for machine learning and deep-learning experiments.
- Comparative analysis of hyperspectral and RGB imaging for sugar content prediction in apple jam, with model performance assessed using standard regression metrics (R^2^, RMSE, MAE).
- Benchmarking of classical machine learning methods (SVM, XGBoost) and deep-learning models (1D ResNet) for regression of sugar concentration levels, with systematic assessment across multiple spatial grid configurations and data-splitting strategies.
- The dataset, code, and trained models are publicly released on GitHub to support reproducibility and further research.

## 2. Materials and Methods

### 2.1. Experimental Setup

**Figure 1:**
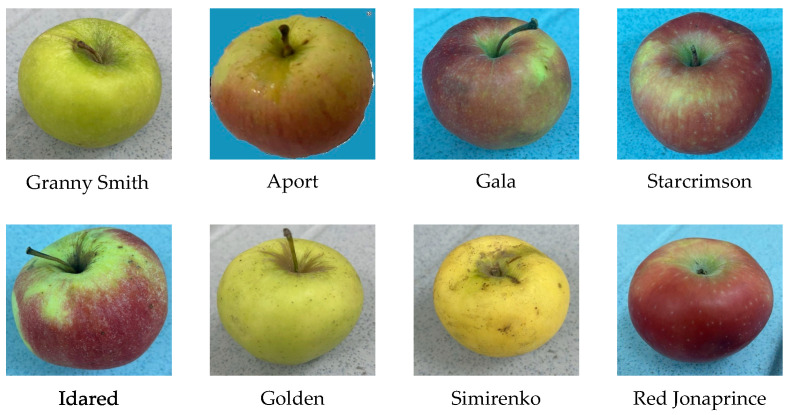
Eight apple cultivars used in this study.

Eight apple cultivars that are common to the Central Asia region, such as Granny Smith, Aport, Gala, Starcrimson, Idared, Golden, Simirenko, and Red Jonaprince, were chosen as a raw material for this study (see Figure 1). Each of these apple cultivars was brought in an amount of 3 kg and then was thoroughly washed, peeled, deseeded, and blended into a homogeneous mass using a conventional food processor. Then, as a next step, sucrose was added in controlled amounts of 5% increments to obtain 11 sugar concentration levels ranging from 25% to 75%. Each mixture was cooked under controlled and consistent conditions until a jam-like texture with uniform consistency and color was obtained. The prepared samples were then distributed on a white dish in three layers of varying thicknesses (0.5 cm, 1 cm, and 2 cm) to obtain hyperspectral images under different conditions.

After the jam samples were prepared, hyperspectral images were acquired using a controlled procedure to ensure consistency and reproducibility across all samples. The imaging system consisted of a portable hyperspectral camera, Specim IQ (Specim, Spectral Imaging Ltd., Oulu, Finland) [40], which operates at a wavelength of 400–1000 nm in the VNIR range and collects data in 204 spectral ranges with a spectral resolution of approximately 3 nm. The camera features a spatial resolution of 512 × 512 pixels and a built-in lens with a diameter of 18 mm, optimized for close-up shooting.

**Figure 2:**
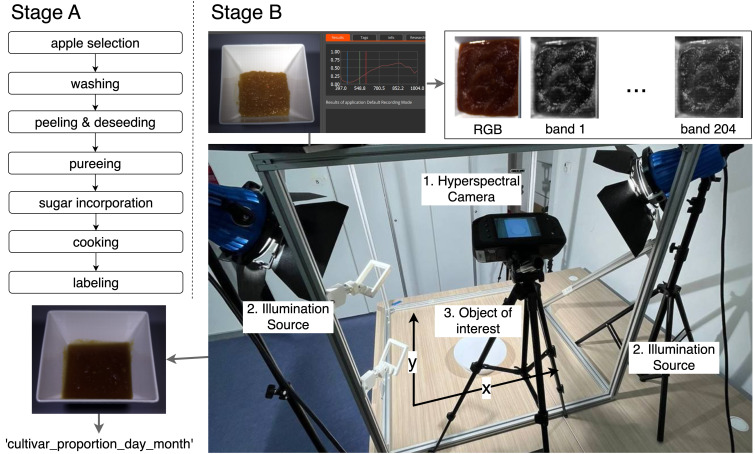
Sample preparation (Stage A) and imaging setup (Stage B) used to acquire RGB and hyperspectral data (204 bands).

To ensure uniform illumination, two 50 W halogen light sources were placed symmetrically at an angle of 45 degrees on both sides of the camera at a height of 50 cm from the sample surface. This configuration minimizes shadows and specular reflections while ensuring uniform illumination throughout the field of view. The hyperspectral camera was mounted on a fixed tripod and positioned directly above the sample to obtain an image from top to bottom, and then shifted at an angle of 45 degrees to obtain an image from the side to capture additional variability of the surface and geometric dimensions (see Figure 2).

The images were taken from three distances: 20 cm, 30 cm, and 40 cm, to ensure variability of spatial resolution and perspective. Radiometric calibration was performed before each imaging session using the integrated procedures provided by Specim IQ. This included capturing a white reference image using the white dish where the jam was placed, and a dark reference image by closing the camera shutter. The calibration data were then used to compute pixel-wise reflectance values R according to the following equation:

(1) $$R=\frac{I-D}{W-D}$$

where I is the raw intensity image, D is the dark reference, and W is the white reference. This correction accounts for sensor noise and illumination nonuniformity, ensuring that the resulting reflectance spectra are consistent across different imaging sessions.

The imaging procedure was carried out for all eight apple cultivars, across eleven sugar concentration levels, and three jam thicknesses (0.5 cm, 1.0 cm, and 2.0 cm). For the 0.5 cm thickness, images were acquired from six different angles, whereas for the 1.0 cm and 2.0 cm thicknesses, images were captured from seven angles. As a result, 1760 hyperspectral images were acquired.

### 2.2. Data Acquisition and Preprocessing

After all the images were acquired, the hyperspectral data, consisting of multiple files generated by Specim IQ per capture, were extracted. For each sample, the .hdr and .dat files representing a hyperspectral cube were isolated as the primary source of reflectance data.

To identify and isolate the jam-covered area from the background, a binary mask was constructed for each image using the spectral angle mapper (SAM) algorithm. This method computes the angular similarity between each pixel’s spectral vector p and a predefined reference spectrum r, obtained from a homogeneous jam region. The spectral angle θ is defined as:

(2) $$\theta (p,r)=\cos^{-1}\left(\frac{p*r}{\left\|p\right\|,\left\|r\right\|}\right)$$

**Figure 3:**
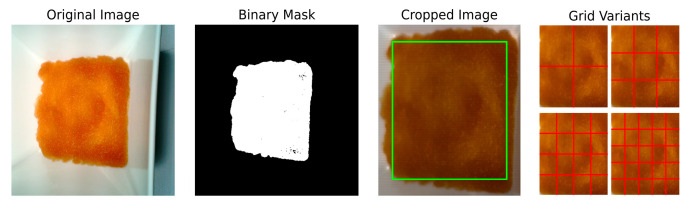
Preprocessing stages of apple jam images.

A threshold of 0.2 radians was applied, and pixels satisfying this criterion were considered part of the jam. The smallest bounding box enclosing these pixels defined the region of interest (ROI), and each image was subsequently cropped to this ROI, the example of which is enclosed in a green rectangle in Figure 3.

While the ROI extraction effectively isolated the jam-covered areas, some cropped images still contained irrelevant visual information, such as the edges of dishes or the background. To minimize this noise and maximize the presence of jam content, a 10% margin was applied from each side of the cropped images prior to subdivision.

Then, each cropped hyperspectral image was subdivided into multiple grids (2 × 2, 3 × 3, 4 × 4, and 5 × 5) to investigate how varying the level of spatial subdivision would affect model learning. These were compared against the original cropped images without any grid applied (1 × 1), allowing for a systematic evaluation of spatial resolution versus predictive performance. This strategy not only expanded the dataset but also aimed to identify the most effective grid configuration for improving generalization in the context of a relatively limited sample size of 1760 full images. An illustration of the applied grid configurations is provided in Figure 3.

Each subregion was processed independently in the following steps. For each of the generated subregions, segmented pixels were used to compute a mean spectral vector, which was then normalized to unit length. This resulted in a compact and consistent feature representation for every jam region.

### 2.3. Statistical Analysis of the Dataset

The finalized dataset consisted of 1760 cropped hyperspectral images of apple jam prepared from eight cultivars native to Central Asia across eleven sugar concentration levels: 25%, 30%, 35%, 40%, 45%, 50%, 55%, 60%, 65%, 70%, 75%. Data were stored in tabular comma-separated values (CSV) format, where each row corresponds to one subregion. In total, five separate datasets were generated based on the grid configurations (1 × 1, 2 × 2, 3 × 3, 4 × 4, and 5 × 5), each representing a different level of spatial subdivision. In addition to the normalized spectral features, each record includes associated metadata: sugar content, apple cultivar label, original image index, and grid position.

To prevent data leakage and evaluate model generalization, we conducted two separate data splitting experiments. In the first experiment, we employed a cultivar-based strategy: out of eight apple cultivars, six (75%) were used for training, one (12.5%) for validation, and one (12.5%) for testing. This strict split ensured that the model was evaluated on completely unseen apple types, allowing us to assess its ability to generalize to novel cultivars. This strategy was consistently applied across all five grid configurations to examine how spatial resolution affects prediction accuracy.

In the second experiment, as illustrated in Table 2, we adopted a sugar concentration- based split. Instead of holding out entire cultivars, we assigned specific sugar concentrations of each apple type exclusively to either the test (≈15.9%) or validation set (≈13.6%), ensuring that those concentrations were absent from the training set (≈70.5%). This arrangement allowed the model to encounter every cultivar during training.

By comparing these splitting approaches, we aimed to understand how the breadth and nature of training data influence the model’s reliability on unseen cases.

### 2.4. Spectral Analysis of Apple Cultivars

**Figure 4:**
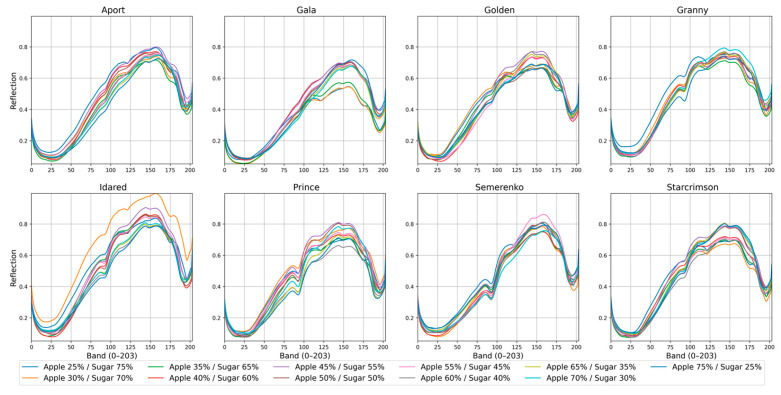
Reflectance spectra (unitless, after white/dark reference calibration) of jam samples prepared from eight apple cultivars (Aport, Gala, Golden, Granny Smith, Idared, Prince, Semirenko, and Starcrimson) across eleven sugar concentration levels (25–75%). Data were acquired using Specim IQ in the 400–1000 nm range with 204 bands.

To provide an overview of the acquired hyperspectral data, the mean reflectance spectra of all eight apple cultivars at eleven sugar concentration levels (25–75%) are presented in Figure 4. Despite the addition of sugar, the overall spectral characteristics remain the same for all varieties, showing a characteristic dip in the 400–500 nm range and reflectance peaks in the 700–900 nm range. Differences among cultivars can still be observed, for example, Idared generally displays higher reflectance across most wavelengths compared to Granny Smith and Gala. The visible differences highlight the potential of hyperspectral sensing to discriminate between apple cultivars and quantify sugar content in apple jams.

### 2.5. Machine Learning

Three regression algorithms were evaluated for predicting sugar content from hyperspectral data: support vector machine (SVM) [41], eXtreme gradient boosting (XG- Boost) [42], and one-dimensional residual network (1D ResNet) [43]. Each model was trained and validated using preprocessed spectral data obtained from five grid configurations (1 × 1, 2 × 2, 3 × 3, 4 × 4, and 5 × 5).

#### 2.5.1. SVM

SVM is a kernel-based supervised learning algorithm commonly used for modeling complex nonlinear relationships in medium-sized regression tasks. In this study, SVM was selected as a classical baseline due to its robustness and strong performance on high-dimensional data such as hyperspectral spectra. We employed the radial basis function (RBF) kernel, which is well-suited for capturing nonlinearity in spectral features [44]. Prior to training, all input vectors were scaled to the [0, 1] range using MinMax normalization to ensure numerical stability.

A grid search was conducted to identify optimal hyperparameters. The best-performing configuration was found to be C = 110, ε = 0.1, and γ = 0.01, based on validation performance. These values provided a good balance between model flexibility and generalization ability.

#### 2.5.2. XGBoost

XGBoost is a gradient-boosted decision tree algorithm widely recognized for its efficiency, scalability, and strong predictive performance on structured tabular data. In this study, XGBoost served as a classical ensemble-based baseline to model nonlinear dependencies in spectral features. Prior to training, all input features were scaled to the [0, 1] range using MinMax normalization. The training data were converted into DMatrix objects for optimized handling within the XGBoost framework.

The model was configured with a learning rate of 0.10, a maximum tree depth of 5, and a column subsampling ratio set to 0.95 to reduce overfitting. Training was conducted for up to 400 boosting rounds, with early stopping applied if the validation root mean square error (RMSE) did not improve for 40 consecutive rounds. The objective function was set to squared error regression.

##### D ResNet

The 1D ResNet used in this study was specifically implemented to handle sequential spectral inputs. The architecture begins with a custom-padded 1D convolutional layer, followed by 8 residual blocks. Each block consists of two convolutional layers with batch normalization and ReLU activation, along with dropout layers. Downsampling is applied every second block, and the number of filters doubles at regular intervals, allowing the net- work to progressively increase feature dimensionality while reducing temporal resolution. This enables the model to capture both fine-grained and high-level spectral patterns. The network concludes with global average pooling and a fully connected regression head.

The model was implemented in PyTorch and trained using the Adam optimizer with a fixed learning rate of 10^−3^ and weight decay of 10^−4^. The loss function used was the mean absolute error (MAE), and training was limited to 30 epochs, with early stopping based on validation performance. If the validation MAE did not improve for 5 consecutive epochs, training was halted.

Before training, all spectral inputs were normalized to the [0, 1] range using MinMax scaling, and sugar content values were likewise rescaled. Data were fed into the net-work using PyTorch’s ‘DataLoader’, with shuffling applied during training. Experiments were conducted using multiple batch sizes (32, 64, 128, and 256) to evaluate the model’s sensitivity to input resolution and training dynamics.

## 3. Results

The models were evaluated on five grid configurations: 1 × 1, 2 × 2, 3 × 3, 4 × 4, and 5 × 5. Performance was assessed using the coefficient of determination (R^2^), RMSE, and MAE. All models were trained on a Lenovo Legion 5 15ARH05H laptop equipped with an NVIDIA GeForce GTX 1660 Ti GPU. Deep learning experiments were conducted in PyTorch (v2.7.0+cu118) with CUDA (v11.8) acceleration, using 1D ResNet models. Each configuration was trained for up to 30 epochs with early stopping (patience = 5), although convergence was typically achieved within 10–15 epochs. Training time ranged from approximately 5 min (e.g., 1 × 1 grid, batch size 256) to about 1 h (e.g., 5 × 5 grid, batch size 32). The extended runtime observed for the 5 × 5 grid with a batch size of 32 reflects the combined effect of subdividing images into finer grids that created more data segments, and smaller batch sizes which required more iterations to complete each epoch. To ensure stability, each setup was repeated across 5 independent runs to account for training variability and to obtain stable estimates of model performance.

Classical models were trained on a CPU using the same laptop without GPU accel- eration. SVM training completed in under one minute per configuration, while XGBoost required slightly longer due to its iterative tree-building process and early stopping. No parallelization or GPU-based acceleration was applied for these models, and experiments were run using a single CPU thread.

These differences in runtime highlight the trade-off between model complexity and computational demand, illustrating that deep neural networks achieve high representational power at the cost of increased training time, whereas classical models remain lightweight and efficient under CPU-only conditions.

### 3.1. Experiment with RGB Images

To evaluate the limitations of RGB imaging for predicting sugar content, models were trained exclusively on RGB data using a concentration-based split across grid sizes. As summarized in Table 3, all models exhibited substantially lower accuracy, with the best CNN result reaching only R^2^ = 0.22, while XGBoost and SVM achieved similarly modest values (R^2^ = 0.21 and R^2^ = 0.18, respectively). Furthermore, an increase in the grid size consistently degraded the performance of all models, likely because finer spatial subdivision broke the data into patches too small for already limited spectral information to retain global patterns, which in turn emphasized noise and local fluctuations, thereby limiting the models’ ability to capture meaningful spectral-spatial relationships.

These systematically low results indicate that RGB signals, limited to three broad channels, are insufficient for detecting biochemical variations, such as sugar concentrations, regardless of their spatial subdivision. The results highlight the necessity of hyperspectral imaging (HSI) for this application.

### 3.2. Cultivar-Based Data Splitting on HSI

The best regression performance was obtained by the ResNet model on the 4 × 4 grid with batch size 64, with an R^2^ of 0.948, RMSE of 3.622, and MAE of 2.764 (see Table 4). Other top-performing configurations included 5 × 5 with a batch size of 64 (R^2^ = 0.944) and 4 × 4 with a batch size of 32 (R^2^ = 0.943), illustrating that generally, moderate spatial resolution (4 × 4 and 5 × 5) proved most beneficial, as finer subdivisions allow the network to capture localized spectral-spatial patterns. Conversely, smaller batch sizes, such as 32 and 64, generally performed better, whereas batch size 256 tended to perform worse. This effect was particularly apparent for the 1 × 1 grid, where a batch size of 256 resulted in a significantly lower mean R^2^ of 0.292, indicating underfitting when trained with low data variation, reflecting the inability of deep models to extract robust spectral-spatial representations under such constrained conditions.

The SVM model showed strong and stable performance across all grid sizes, reaching its best results at the 4 × 4 configuration with R^2^ = 0.938, RMSE = 3.940, and MAE = 3.107. This stability highlights SVM’s robustness to data partitioning. Similarly, as shown in Table 4, XGBoost performed best at the 4 × 4 grid (R^2^ = 0.9344, RMSE = 4.048, MAE = 3.173), with competitive scores across all configurations.

Overall, the 4 × 4 spatial subdivision consistently yielded the best outcomes across all methods. ResNet achieved the strongest individual result, while SVM and XGBoost provided more uniform performance across grid sizes. These findings suggest that utilizing spatial subdivision leads to improved regression accuracy and that neural models, such as ResNet, could have greater potential with proper tuning.

### 3.3. Sugar Concentration-Based Data Splitting on HIS

Overall, the concentration-based splitting approach resulted in improved regression performance across all models compared to the cultivar-based split, confirming that exposure to partial information from all cultivars allows models to generalize more effectively across sugar concentration levels. According to Table 5, the highest performance was achieved by the ResNet model on the 5 × 5 grid with batch size 64, reaching an R^2^ of 0.962, RMSE of 2.754, and MAE of 2.129. Other strong configurations included 4 × 4 and 3 × 3 with R^2^ values of 0.955 and 0.953, respectively. These results reaffirm the benefits of spatial subdivision and suggest that when partial information about all cultivars is available, the model can generalize more easily across sugar levels.

Interestingly, while the SVM model also showed strong performance, peaking at R^2^ = 0.958 on the 5 × 5 grid (see Table 5), XGBoost exhibited noticeably lower performance than in the cultivar-based experiment. Its best R^2^ was 0.889 on the 2 × 2 grid, and in contrast to the other models, increasing spatial resolution did not lead to noticeable performance gains, which suggests that kernel-based methods can reliably exploit spectral features regardless of grid configuration. In XGBoost, the lack of benefit from increased spatial resolution suggests that gradient-boosted trees are less adept at capturing subtle spectral-spatial patterns compared to ResNet and SVM.

**Figure 5:**
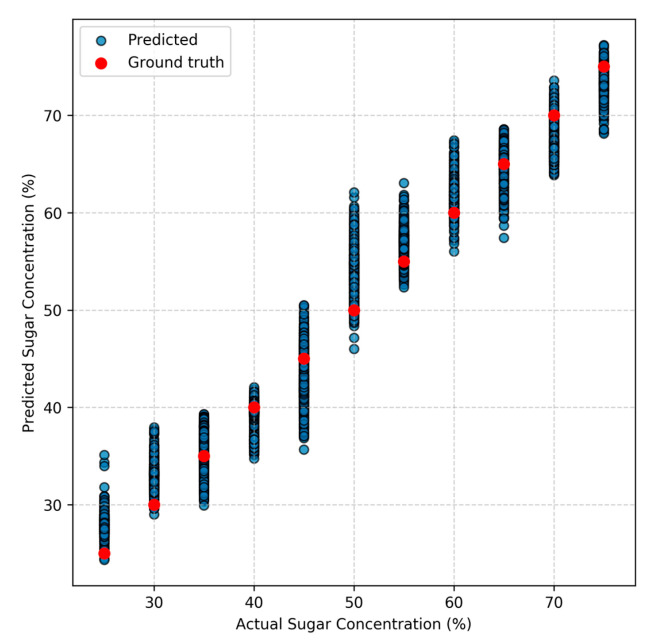
Scatter plot of predicted versus actual sugar concentrations for the 5 × 5 grids test set using the 1D ResNet model. Each blue point represents one prediction, and the red points indicate the ground truth sugar concentration levels.

Among all models, ResNet showed the most significant gain from this split strategy, indicating its ability to capitalize on subtle differences in the data. Both ResNet and SVM benefited from higher spatial resolution, with 5 × 5 and 4 × 4 grids yielding the strongest results. Figure 5 presents a scatter plot of predicted versus actual sugar concentrations on the test set. The close clustering of predicted points around the red ground truth points demonstrates that the 1D ResNet model provides highly accurate predictions across concentration levels.

## 4. Discussion

This study investigated the potential of combining hyperspectral imaging with machine learning as a non-invasive method for predicting sugar content in processed apple jam. Traditional techniques, although accurate, are often destructive and time-consuming, making them less suitable for high-throughput or real-time applications [45]. By using spectral data captured from a portable VNIR camera and applying both classical and deep learning models, we aimed to evaluate a fast and scalable alternative for quality assessment in processed foods.

The choice of hyperspectral imaging was not arbitrary, but necessary, as conventional RGB images provide only three broad visible bands (red, green, and blue) with limited spectral resolution. The large gap in predictive power comes from the difference in both the number and range of spectral bands. RGB images capture only three broad channels in the visible spectrum (around 400–700 nm), which provide limited information about the sample. Hyperspectral imaging, by contrast, records 204 narrow, contiguous bands that extend into the near-infrared region up to 1000 nm. This wider and more detailed coverage enables the system to detect subtle variations in reflectance that are directly influenced by the chemical composition of the jam, including differences related to sugar content. The results in Table 3 confirm that hyperspectral imaging is indispensable for reliable sugar content estimation in processed products.

Previous studies have applied HSI to predict sugar content in raw agricultural products, including tomatoes (R^2^ = 0.90, MSE ≈ 0.018 °Brix) [30], kiwifruit (R^2^ = 0.95, RPD = 3.26) [32], and peaches (R^2^ = 0.92, RMSE ≈ 0.67 °Brix) [34]. Importantly, these earlier studies evaluated model performance primarily under random data splits, which do not fully test a model’s ability to generalize to unseen cultivars or varying sugar levels. In contrast, our work introduced two more rigorous partitioning strategies: a cultivar-based split, in which entire apple varieties were excluded from training, and a sugar concentration-based split, in which ranges of concentration values were held out. Under these stricter scenarios, our best-performing ResNet model achieved R^2^ = 0.948 (cultivar split) and R^2^ = 0.962 (concentration split), with MAE values of about 2.1 °Brix. These results are competitive with, and in some cases exceed, those reported for raw fruits, demonstrating that hyperspectral learning is also effective for processed products, where cooking, homogenization, and the addition of ingredients lead to different optical and chemical properties compared to fresh produce. The incorporation of cultivar- and concentration-based splits provides a more rigorous benchmark for evaluating generalization compared to random partitions, making this study one of the first to report results on processed jams and to apply such splitting strategies in HSI-based food quality assessment.

Under the cultivar-based splitting strategy, where entire apple types were held out from training, the one-dimensional ResNet model consistently outperformed classical methods, achieving a peak R^2^ of 0.948. Its strong performance is attributed to its ability to capture complex spectral features through deep residual connections. Notably, this splitting approach revealed meaningful and consistent trends across all models. Specifically, the 4 × 4 spatial grid yielded the best results overall, while the 5 × 5 grid performed slightly worse, suggesting that excessive spatial subdivision may dilute the signal with noise. Smaller grids, such as 1 × 1 and 2 × 2, often underperformed, likely due to insufficient spatial diversity. These results suggest an optimal balance near the 4 × 4 configuration, where each patch retains sufficient relevant information while minimizing noise interference. For ResNet, batch size also influenced its performance: moderate values often resulted in more stable and accurate learning outcomes, while the largest size (256) sometimes led to unstable training or reduced accuracy, particularly with smaller grids. These observations make cultivar-based splitting a valuable benchmark, as it presents a more challenging yet interpretable setting for evaluating model behavior in relation to architectural and input design choices.

In contrast, the concentration-based splitting strategy produced higher overall performance across models. ResNet reached a peak R^2^ of 0.962, while SVM achieved up to 0.958. However, the trends were less consistent. SVM and XGBoost showed nearly uniform performance across all grid sizes, lacking the clear benefit from increased spatial resolution observed under the cultivar split. In particular, XGBoost underperformed relative to its results in the cultivar-based setting, suggesting that it may benefit more from encountering entirely unseen samples rather than partial exposure across types. Only 1D ResNet maintained a meaningful response to spatial subdivision, continuing to improve with finer grids and showing its capacity to learn from localized spectral structures even under less strict splitting.

When comparing the two strategies, it is evident that while the concentration-based approach led to better accuracy, it is a less strict test of the model’s ability to generalize. Since the model sees every apple type during training, it can rely on shared visual and spectral features to estimate sugar content, making the task easier. On the other hand, cultivar-based splitting presents a more realistic and robust scenario where the model must predict sugar content for apple types it has never seen before. Despite slightly lower performance, this setup revealed more meaningful trends, such as consistent improvements with finer grid sizes and more stable performance at intermediate batch sizes. The 4 × 4 grid consistently yielded the best results across models, while the 5 × 5 grid performed slightly worse, suggesting that excessive subdivision may introduce noise or reduce the useful signal.

**Figure 6:**
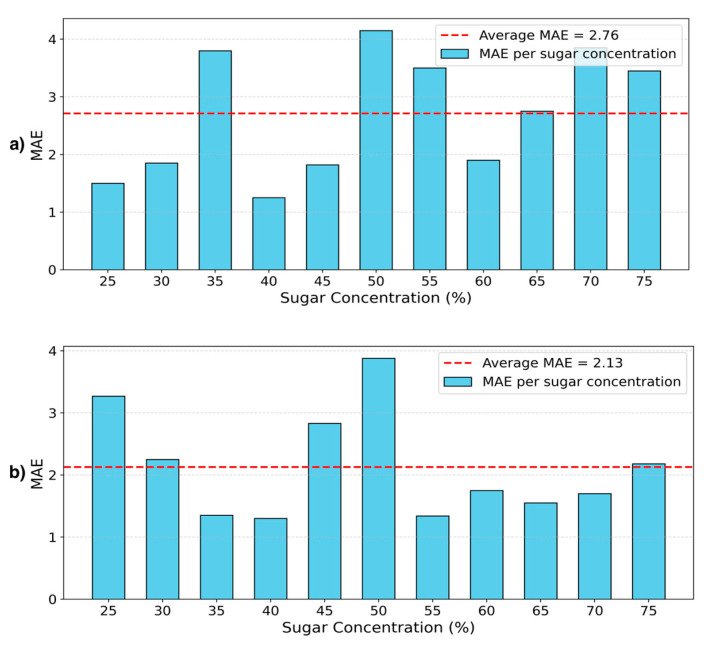
MAE per class for the best-performing ResNet model under two data-splitting strategies: (**a**) cultivar-based splitting and (**b**) sugar concentration-based splitting.

This contrast is further illustrated in Figure 6, where MAE per class is shown for the best-performing ResNet model under both splitting strategies. Under the concentration- based split, Figure 6b shows that MAE is generally lower and more consistent across sugar levels, indicating that the model benefits from shared visual and spectral patterns seen during training. In contrast, the cultivar-based split results in higher average MAE and greater variability, revealing the model’s sensitivity to truly unseen data and underscoring the difficulty of this generalization setting (see Figure 6a).

While the results demonstrate strong potential for applying hyperspectral imaging and machine learning in processed food analysis, the study was subject to several limitations that should be considered. One limitation observed was that, although most hyperspectral images were of high quality, some showed minor inconsistencies in jam placement or camera angle, which introduced variability in the size and alignment of the cropped regions used for analysis. Another limitation involved occasional reflectance artifacts caused by strong lighting, where overexposed areas were incorrectly represented as dark regions due to an imperfect white reference. These cases were limited in number and did not noticeably affect overall model performance. Nonetheless, future work could improve robustness by implementing a more standardized imaging setup and calibration process. Finally, the scope of the study was limited to a single product type, and further research is needed to assess whether the proposed approach generalizes well to other processed food items with varying textures, compositions, and optical characteristics.

## 5. Conclusions

In the course of this study, we generated a novel dataset composed of eight apple cultivars, obtained from the most diverse source of apples in the world, comprising 1760 samples. These samples included sugar concentrations ranging from 25% to 75% and were captured at different angles of imaging.

This study demonstrated that hyperspectral imaging, combined with machine learning models, offers a promising non-destructive solution for predicting sugar content in processed apple jam. Among the evaluated methods, the one-dimensional ResNet model consistently achieved the highest predictive accuracy across both data splitting strategies, particularly when applied to moderately segmented spatial regions. While ResNet required longer training times and careful tuning, its strong performance highlights the advantages of deep learning in capturing complex spectral and spatial patterns.

Classical models, such as SVM and XGBoost, although generally less accurate, still produce reliable estimates with significantly lower computational costs and faster training times. These results demonstrate a practical trade-off: deep models offer superior performance for high-precision applications, while classical models may be preferred in settings where speed and simplicity are prioritized.

By focusing on a processed product type that has often been overlooked in previous hyperspectral studies, this work contributes to expanding the applicability of HSI beyond raw products. The proposed approach provides a foundation for developing scalable, real-time quality control tools in food processing environments. Future work may extend these findings to other product types, explore alternative model architectures, and evaluate robustness under industrial conditions. Future work may extend these findings beyond sugar content prediction to other product types, alternative model architectures, and evaluations under industrial conditions. Moreover, hyperspectral imaging could be further applied to critical domains such as food authenticity and food adulteration detection, which are essential for safeguarding product integrity and consumer trust. In this way, HSI has the potential to serve not only as a tool for compositional quality assessment but also as a robust approach for addressing authenticity verification and adulteration monitoring across diverse food systems.

## Figures and Tables

**Table 1:** Comparative analysis of existing solutions.

Literature	Product	Type	Deep Learning	Classical ML	Target Trait(s)
[29]	Potato	Raw	✗	✓	SSC
[30]	Tomato	Raw	✓	✓	SSC, Firmness
[31]	Grapevine	Raw	✓	✓	Water status
[32]	Kiwi	Raw	✗	✓	SSC, Firmness
[33]	Strawberry	Raw	✓	✓	SSC, Maturity
[34]	Peach	Raw	✓	✓	SSC
[35]	Apple	Raw	✗	✓	SSC
[36]	Apple	Raw	✓	✓	SSC
[37]	Apple	Raw	✗	✓	SSC, Firmness
[38]	Cheese	Processed	✗	✓	Fat content
[39]	Milk Powder	Processed	✗	✓	Melamine
**This work**	Apple Jam	Processed	✓	✓	SSC

**Table 2:** Distribution of sugar concentration values across apple jam types. * * * * = Test, * * * * = Validation.

Apple Jam Type	Sugar Concentrations in %										
aport	25	30	35	40	45	50	55	60	65	70	75
gala	25	30	35	40	45	50	55	60	65	70	75
golden	25	30	35	40	45	50	55	60	65	70	75
granny	25	30	35	40	45	50	55	60	65	70	75
prince	25	30	35	40	45	50	55	60	65	70	75
idared	25	30	35	40	45	50	55	60	65	70	75
simirenko	25	30	35	40	45	50	55	60	65	70	75
starcrimson	25	30	35	40	45	50	55	60	65	70	75

**Table 3:** Performance of models trained on RGB images under concentration-based splitting across grid sizes.

Model	Grid Size	R^2^	RMSE	MAE
1D ResNet	1 × 1	0.22	13.96	11.48
	2 × 2	0.14	14.59	11.84
	3 × 3	0.11	14.87	12.18
	4 × 4	0.08	15.10	12.24
	5 × 5	0.06	15.28	12.28
SVM	1 × 1	0.18	14.27	11.81
	2 × 2	0.14	14.61	12.11
	3 × 3	0.13	14.71	12.24
	4 × 4	0.12	14.81	12.31
	5 × 5	0.11	14.83	12.32
XGBoost	1 × 1	0.21	13.98	11.78
	2 × 2	0.18	14.29	11.87
	3 × 3	0.17	14.39	11.98
	4 × 4	0.14	14.58	12.13
	5 × 5	0.14	14.61	12.18

**Table 4:** Performance of 1D ResNet, SVM, and XGBoost across grid size configurations under cultivar-based splitting.

Model	Grid Size	R^2^	RMSE	MAE
1D ResNet	1 × 1	0.92	4.47	3.60
	2 × 2	0.92	4.54	3.66
	3 × 3	0.90	4.18	3.31
	4 × 4	**0.95**	**3.62**	**2.76**
	5 × 5	0.94	3.73	3.01
SVM	1 × 1	0.90	4.95	4.04
	2 × 2	0.90	5.13	4.17
	3 × 3	0.90	4.95	3.83
	4 × 4	**0.94**	**3.94**	**3.11**
	5 × 5	0.92	4.42	3.59
XGBoost	1 × 1	0.84	6.27	4.71
	2 × 2	0.91	4.63	3.65
	3 × 3	0.92	4.42	3.50
	4 × 4	**0.93**	**4.05**	**3.17**
	5 × 5	0.92	4.37	3.44

**Table 5:** Performance of 1D ResNet, SVM, and XGBoost across grid size configurations under concentration-based splitting.

Model	Grid Size	R^2^	RMSE	MAE
1D ResNet	1 × 1	0.91	4.26	3.36
	2 × 2	0.92	4.02	3.08
	3 × 3	0.95	3.20	2.40
	4 × 4	0.95	3.13	2.42
	5 × 5	**0.96**	**2.75**	**2.13**
SVM	1 × 1	0.96	2.98	2.40
	2 × 2	0.96	2.99	2.52
	3 × 3	0.96	2.98	2.47
	4 × 4	0.96	2.92	2.37
	5 × 5	**0.96**	**2.92**	**2.39**
XGBoost	1 × 1	0.89	4.74	3.67
	2 × 2	0.89	4.72	3.65
	3 × 3	0.89	4.73	3.67
	4 × 4	**0.89**	**4.76**	**3.70**
	5 × 5	0.89	4.76	3.69

## Data Availability

All data, models, and usage instructions are publicly available in our GitHub repository (https://github.com/IS2AI/HSI_Apple_Jam/tree/main, accessed on 10 August 2025) and on our Hugging Face dataset page (https://huggingface.co/datasets/issai/Apples_HSI, accessed on 10 August 2025).

## Abbreviations

The following abbreviations are used in this manuscript:

HSI	Hyperspectral imaging
VNIR	Visible to near-infrared
TSS	Total soluble solids
SSC	Soluble solids content
PLSR	Partial least squares regression
ROI	Region of interest
CSV	Comma separated values format
SVM	Support vector machine
XGBoost	eXtreme gradient boosting
1D ResNet	One-dimensional residual network
MAE	Mean absolute error
